# Supplementary material for: Global arthropod beta-diversity is spatially and temporally structured by latitude
Source: Commun Biol. 2024 May 8;7:552. doi: 10.1038/s42003-024-06199-1 (PMC11078949; doi:10.1038/s42003-024-06199-1)

Supplementary online material

Supplementary Fig. 1. Sample diversity across space and time. Regional (indicated in subplot headings) variation in BIN richness (y-axes) vs absolute latitude (top), mean distance to all other sampling locations (middle) and month of sampling (bottom). Boxplots show the distribution of the data with the whiskers representing the first and third quartiles, and the points shown for samples outside this range. Colors correspond to those used to differentiate regions in Figure 1. N=2,412.

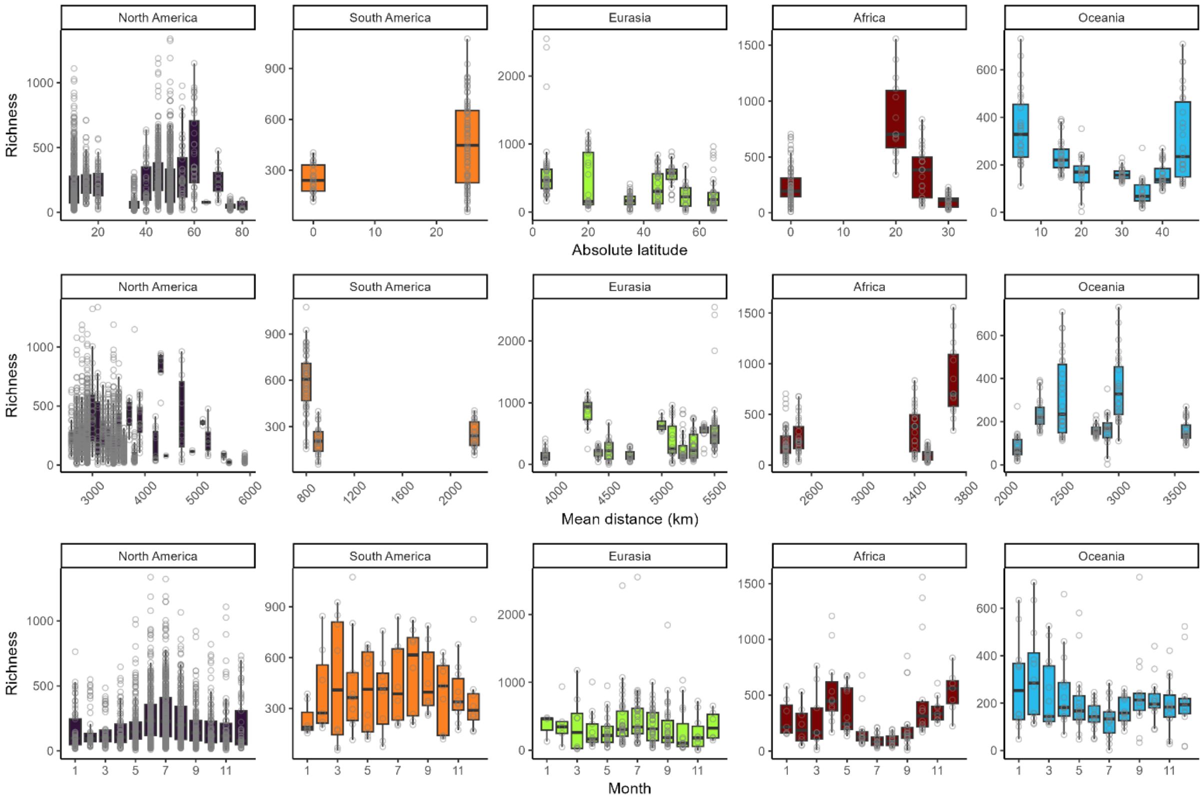

Supplementary Fig. 2. Beta-diversity and its compositions by region. Regional (indicated in subplot headings) variation in Beta-diversity (Jaccard dissimilarity) (y-axes) and its compositional components richness difference (RichDiff) and replacement (Repl). Each point represents a unique site pairing within the respective regional group. N=2,910,078

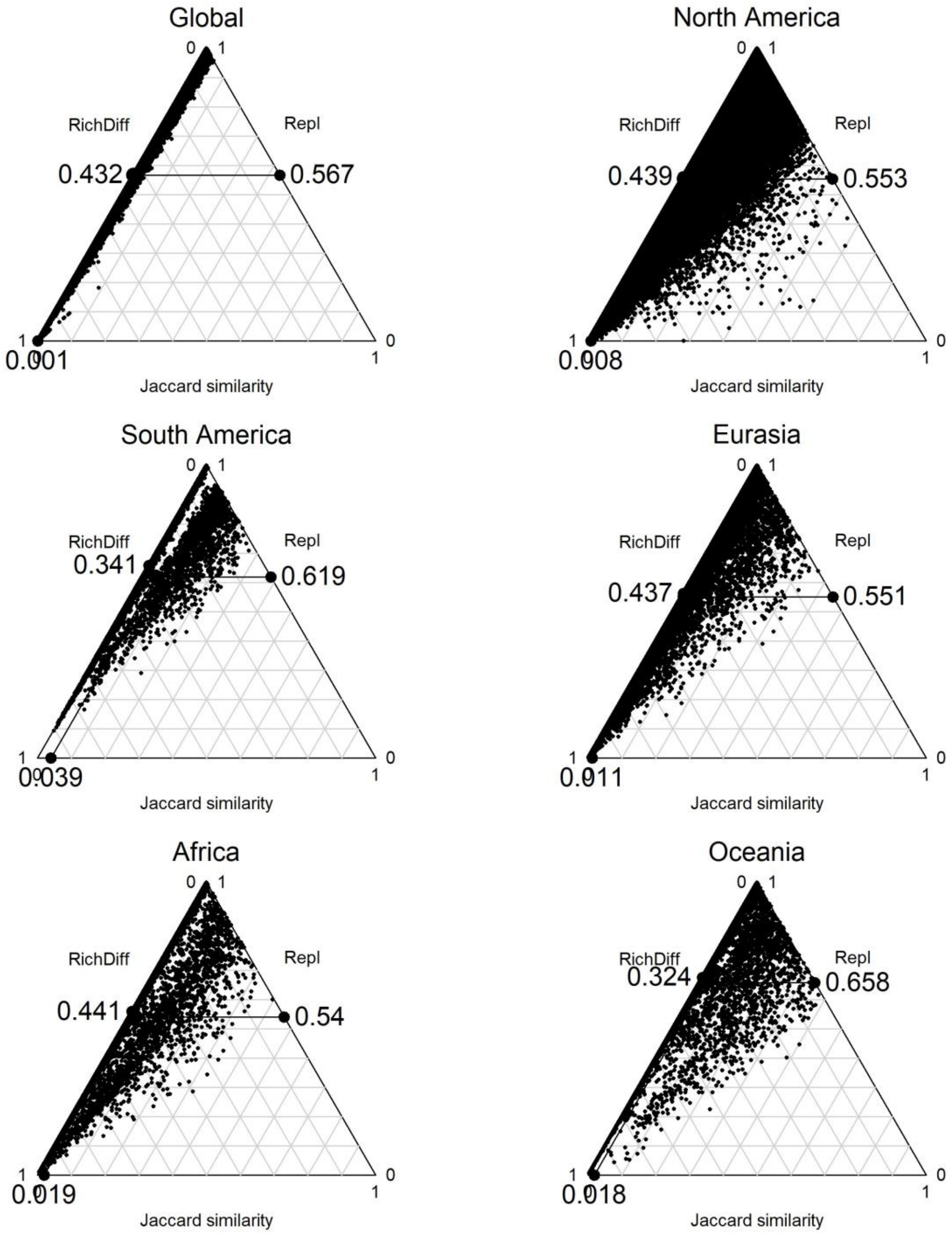

Supplementary Fig. 3. Total Beta-diversity patterns. Impacts of pairwise distance in space and latitude (top 6 plots) and pairwise distance in time and latitude (bottom 6 plots) on total Beta-diversity. This figure shows the beta-diversity, plotted from the fitted values of a linear model of the metric in question as a function of distance in space, distance in time (here set to zero), mean absolute latitude, and the interactions mean latitude × distance in space and mean latitude × distance in time (see Table 1 for statistical significances). Note the differences in the scaling of axes among the graphs.

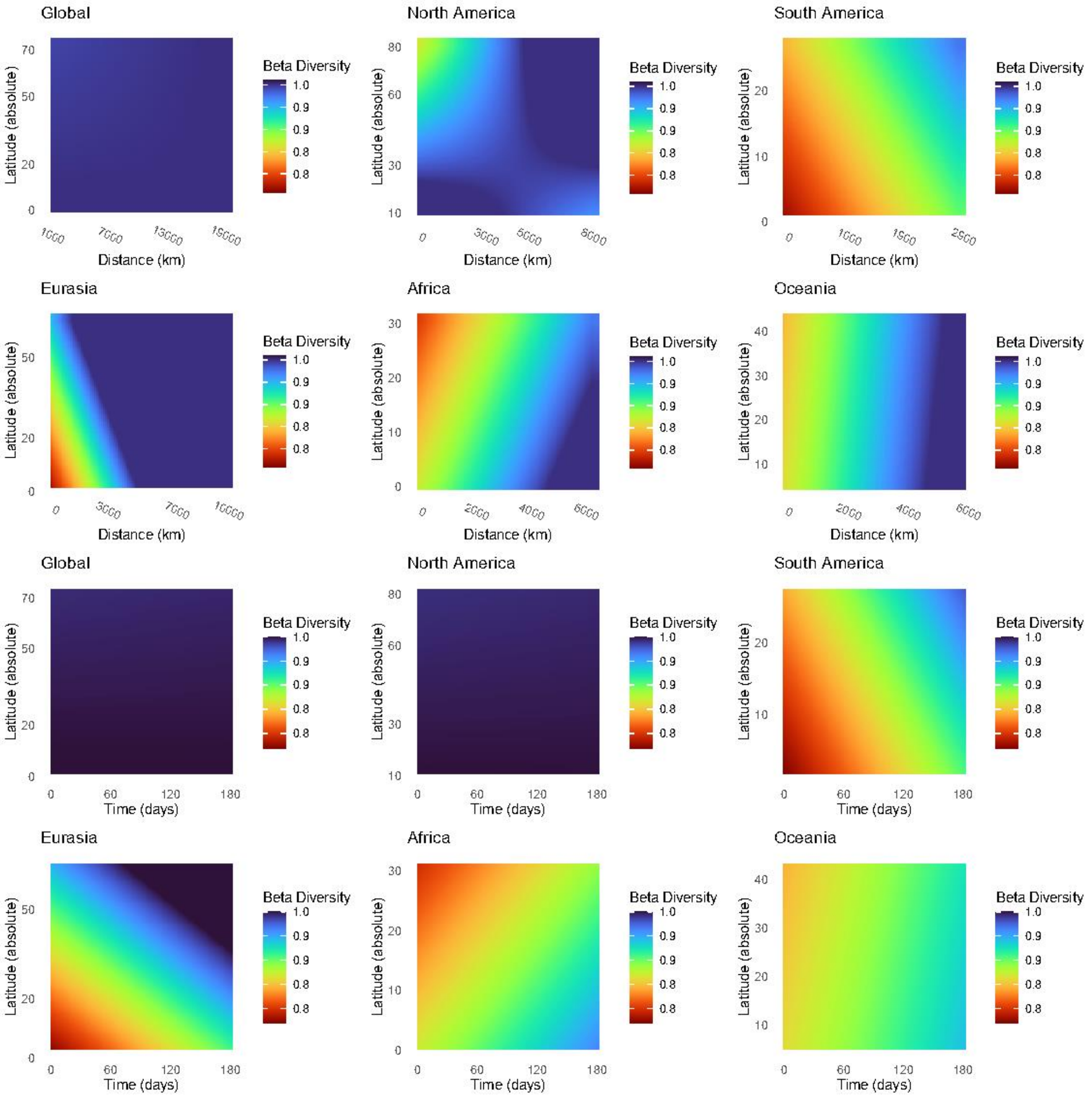

Supplementary Fig. 4. Species richness across habitat types. Each point is a unique trap event with each color indicating the geographic region corresponding with the colors shown in Figure 1. N=2,412

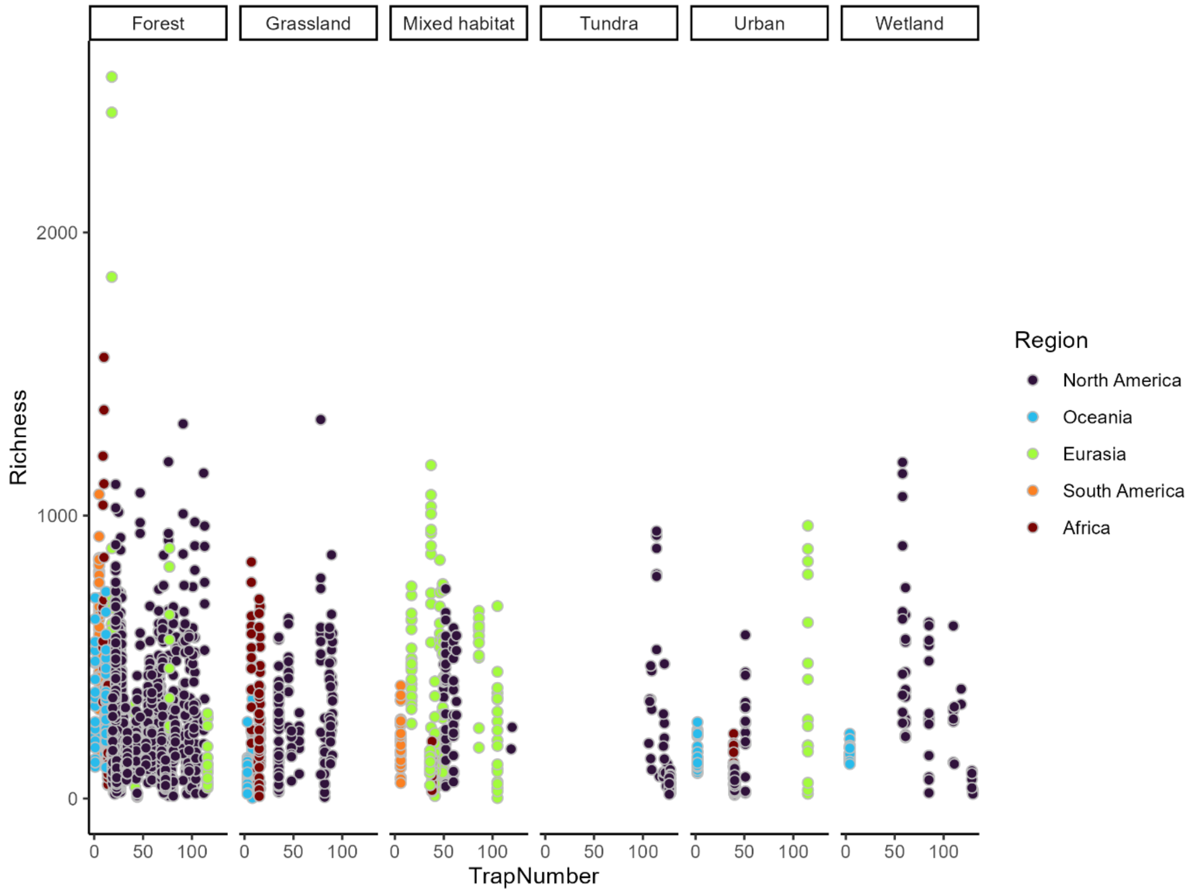

Supplement: Supplementary file 2 — Supplementary Material [file 42003_2024_6199_MOESM2_ESM.pdf]
