# Supplementary material for: Global arthropod beta-diversity is spatially and temporally structured by latitude
Source: Commun Biol. 2024 May 8;7:552. doi: 10.1038/s42003-024-06199-1 (PMC11078949; doi:10.1038/s42003-024-06199-1)
Supplement: Supplementary file 3 — Description of Additional Supplementary Files [file 42003_2024_6199_MOESM3_ESM.pdf]

Description of Additional Supplementary Files

File name: Supplementary Data 1

Description: Meta data containing site level data. Provided are site codes and names, Country where the site is located, Region as described in the main text, The collectors that lead the sampling efforts, The latitude and longitude of the location, The number of trap events (sample collections) taken from the stie, the start and end dates for when the sampling location was used for trap event and sample collection.

File name: Supplementary Data 2

Description: Meta data containing trap event data. Provided are the trap event ID, Date of the trapping event, the Country the event took place in. The latitude and longitude, The site number to link with the source data, the associated habitat, landuse and region as depicted in the main text.

File name: Supplementary Data 3

Description: The source data file for all individual arthropods included in the study. Provided are the Trap event ID, BIN ID, Order level taxonomy, Family level taxonomy and site number ID.
